# Deep learning ­– promises for 3D nuclear imaging: a guide for biologists

**DOI:** 10.1242/jcs.258986

**Published:** 2022-04-14

**Authors:** Guillaume Mougeot, Tristan Dubos, Frédéric Chausse, Emilie Péry, Katja Graumann, Christophe Tatout, David E. Evans, Sophie Desset

**Affiliations:** 1 Université Clermont Auvergne, CNRS, Inserm, GReD, F-63000 Clermont-Ferrand, France; 2 Department of Biological and Molecular Sciences, Faculty of Health and Life Sciences, Oxford Brookes University, Oxford OX3 0BP, UK; 3 Université Clermont Auvergne, Clermont Auvergne INP, CNRS, Institut Pascal, F-63000 Clermont–Ferrand, France

**Keywords:** 3D nucleus, Deep learning, 3D microscopy images, 3D segmentation, Image dataset, Open source

## Abstract

For the past century, the nucleus has been the focus of extensive investigations in cell biology. However, many questions remain about how its shape and size are regulated during development, in different tissues, or during disease and aging. To track these changes, microscopy has long been the tool of choice. Image analysis has revolutionized this field of research by providing computational tools that can be used to translate qualitative images into quantitative parameters. Many tools have been designed to delimit objects in 2D and, eventually, in 3D in order to define their shapes, their number or their position in nuclear space. Today, the field is driven by deep-learning methods, most of which take advantage of convolutional neural networks. These techniques are remarkably adapted to biomedical images when trained using large datasets and powerful computer graphics cards. To promote these innovative and promising methods to cell biologists, this Review summarizes the main concepts and terminologies of deep learning. Special emphasis is placed on the availability of these methods. We highlight why the quality and characteristics of training image datasets are important and where to find them, as well as how to create, store and share image datasets. Finally, we describe deep-learning methods well-suited for 3D analysis of nuclei and classify them according to their level of usability for biologists. Out of more than 150 published methods, we identify fewer than 12 that biologists can use, and we explain why this is the case. Based on this experience, we propose best practices to share deep-learning methods with biologists.

## Abstract (precis)

**Summary:** Deep-learning methods are introduced for users, highlighting accessible methods for 3D nucleus analysis and encouraging interdisciplinary research between computer scientists and biologists.

## Introduction

The cell nucleus is at the centre of many investigations in biology. Its characterization is, for example, commonly used to describe new genetic functions or to classify tumour cells. Indeed, the nucleus presents a dynamic morphology with a wide range of variations in shape and size, as well as a dynamic content organized into chromosomal domains and nuclear bodies. To link nuclear organization and function, images of nuclei are captured by microscopy and then subjected to 2D or 3D morphometric analysis to quantify their morphology and the position and organization of nuclear domains (Andrey et al., 2010; Poulet et al., 2017, 2015). Many platforms and libraries dedicated to the analysis of images from microscopy have been developed, of which the best known are ImageJ/Fiji (Schindelin et al., 2012), CellProfiler (Carpenter et al., 2006), Imaris (Oxford Instruments; https://imaris.oxinst.com/), Icy (De Chaumont et al., 2012) and, more recently, Ilastik (Berg et al., 2019). They embed an entire series of mathematical methods for image processing that are assembled to form pipelines or plugins. Open-source tools dedicated to the quantitative description of the 3D nucleus include TANGO (Ollion et al., 2013) and NucleusJ (Dubos et al., 2020). Most of these tools require human intervention to reach a sufficient accuracy and become very specialized once configured for a certain image type.

Recent improvements in imaging techniques have significantly shortened the time needed to capture microscopy images, especially for the cell nucleus as it is one of the most studied cell organelles. Consequently, the amount of data generated by biologists has drastically increased, leading them to seek methods that reduce the burden of manual analysis and automate quantitative studies to facilitate large-scale statistical analysis (Eliceiri et al., 2012). Concurrently, computer scientists have developed a plethora of techniques for image and bioimage analysis (Lucas et al., 2021), among which deep learning (DL) stands out for its accuracy in many computer vision problems (von Chamier et al., 2019). Additionally, DL methods almost automatically adapt to new kinds of data, thus requiring much less human intervention.

Unfortunately, this new panacea for bioimaging also has some drawbacks. Most DL methods require a significant amount of manually annotated data to work properly and typically must be trained on large datasets before they can be used. Existing datasets are often not publicly accessible, and creating new ones requires time. DL methods also require a high-performance computer graphics card and use many software libraries (suites of programs) that are complex to set up. Reusing an old version of a high-level DL library is difficult because it depends on lower-level libraries that are frequently revised and often incompatible from one version to another. Finally, DL code is far from simple and usually needs to be configured by an expert. Despite some initiatives to assist users that are not proficient in DL, DL methods are still not easily accessible to biologists (Laine et al., 2021). Thus, the main objective of this Review is to provide biologists with a roadmap for understanding and using DL methods that will also help them to select the most appropriate method for their needs. Particular attention is paid to the accessibility of methods both for end users and developers, as despite the profusion of publications, only a few DL methods are usable.

In this Review, we open the ‘black box’ of DL, explaining its most important elements and how they are shared in biology, as well as detailing accessible and up-to-date DL methods that can be applied to the analysis of the 3D nucleus.

## Opening the DL black box for bioimaging

DL techniques first achieved ‘superhuman’ performance (i.e. exceeding that of a human experimenter) on visual task problems as recently as 2011 (Cireşan et al., 2011), owing to the implementation of convolutional neural networks (CNNs) – a new DL method – on fast graphical processing units (GPUs) and large-scale datasets. CNNs became famous in 2012 by winning several major competitions, including ImageNet 2012 (Krizhevsky et al., 2012). Afterwards, most computer vision scientific communities began to use these new methods, and biomedical image analysts shortly followed. Many of the recent publications in both fields now use CNNs (Moen et al., 2019), with the U-Net model (Ronneberger et al., 2015) a well-known example.

### What is DL and how does it work?

**Fig. 1.**
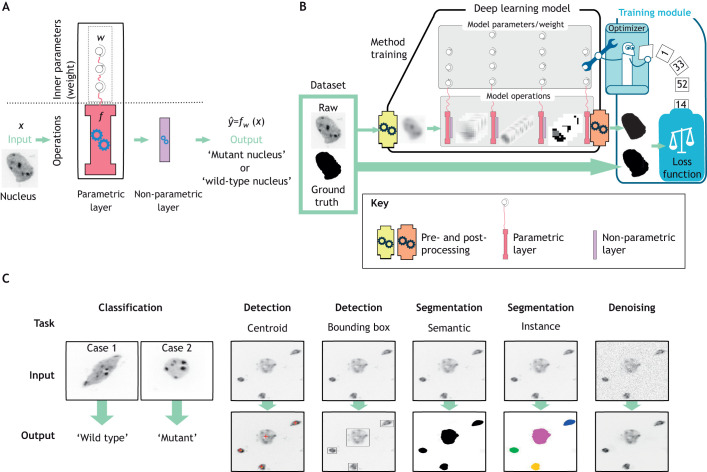
**Basic principles of machine learning methods for image analysis.** (A) Basic units of an ML model. The two-layer model shown here takes a nucleus image as input and decides whether it is a mutant or a wild-type nucleus. The trainable layer (red, parametric layer) is parametrized by three adjustable parameters (weights) that determine its behaviour. The non-trainable layer (purple, non-parametric layer) is not adjusted during training and here applies a fixed threshold to the input. (B) Schematic illustration of a DL method and its training for image analysis. A DL model for segmentation converts a raw image (left) into a binary mask (right). It comprises a pre-processing step (yellow engine), several sets of layers (central grey rectangles) and a post-processing step (orange engine). The output of the model is then transferred into the training module together with the ground truth images. The aim of the loss function (white scale) is to compare the output and ground truth images and to compute the loss value that assesses the performance of the model. The model parameter values are then either increased or decreased by the optimizer (robot), depending on the loss value, and this process is reiterated until a satisfactory loss value is reached. Subsequently, the training module is removed to yield the so-called trained model that can now be used to infer predictions from new images. (C) Typical image analysis tasks for images of nuclei solved by DL methods. From left to right: classification of two nuclei into two categories, wild-type or mutant; detection of the centroids of four nuclei present (red crosses); detection of the bounding boxes around the four nuclei; semantic segmentation of the entire image into a binary mask where nuclei are coloured in black and the background in white; instance segmentation of the four nuclei into four classes (coloured regions) and of the background (white region); denoising of the image.

DL belongs to the broader class of machine-learning (ML) methods. ML is the art of writing a computer program that can learn to recognize or predict patterns. Informally, an ML algorithm can be seen as a black-box machine fed by an input, such as an image, that produces an output, such as a number (Fig. 1A). The machine, called a model, is a set of operations adjustable with inner ‘buttons’, called parameters or weights (*w*), changing the way the output is produced for a given input. Mathematically, a model can be seen as a function *f* with a set of parameters *w* that, given an input *x*, produces an output 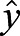, which can be summarized as 
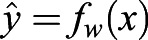
. The performance of the model is then evaluated by the loss function (see Glossary). The loss function takes the model output 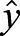 and the expected results, which are called the ground truth (*y*), and compares them by giving a score, 
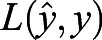
, that represents the performance of the model. In most cases, a loss function gives a positive real number that must be minimized when adjusting the weights. Depending on the loss score, the model parameters are then updated by an algorithm called the optimizer. Computing a loss value and updating the parameters is called training (see Glossary) (Fig. 1B). Among the many existing optimizers (Géron, 2019), the most used is gradient descent. This algorithm can be illustrated by imagining oneself blinded by fog on a hillside that one would like to descend. By feeling the direction of the slope and by moving stepwise in that direction, the valley will be reached. In our ML scope, one position on the ground represents one configuration of the model parameters, the altitude represents the loss value obtained on a set of data and the slope is the so-called gradient. Mathematically, the loss gradient is noted *dL_w_*/*dw* , and the model parameters are updated by subtracting it: 
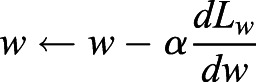
. The hyperparameter *α* (see Glossary) is called the learning rate and represents the size of the step in the direction of gradient. The most used optimizers based on gradient descent are Stochastic Gradient Descent, RMSProp and Adam (Ruder, 2016 preprint).

To introduce DL-specific vocabulary, consider a greatly simplified example: differentiating mutant and wild-type nuclei using 2D images (Fig. 1A). To achieve this, we could compute the weighted sum of all the pixels in an image containing one nucleus and apply a threshold value to obtain a single digit: 1 for a mutant nucleus and 0 for wild type. In this example, the weights in the weighted sum are the parameters. They are randomly initialized and must be adjusted during the training stage; this provides a so-called trainable layer (see Glossary). The user could impose the threshold value applied afterwards; in this case, the thresholding layer is non-trainable. This layer also belongs to the sub-category of activation layer as it imposes a choice by transforming a continuous output to a binary one. This two-layers model has only a single trainable layer and is therefore a shallow ML model. To transform it into a DL model, at least one more trainable layer must be added before or after the existing one. This could be achieved by, for instance, dividing the initial 2D image into 16 equal parts, computing a new weighted sum and applying a threshold to each part, giving us 16 binary values that can then be reordered in a four-by-four image called a feature map (see Glossary). A weighted sum computation and thresholding application can now be reapplied to this image to obtain the final decision. This four-layer model is a DL model because it contains a succession of two trainable layers (see Glossary). The first trainable layer focuses on simple patterns in small local regions of the image, and the associated activation layer can force the model to decide whether each region contains an area of wild-type or mutant nucleus. The second deeper layer treats more complex patterns in the whole image and takes the final decision. DL models thus decompose their input by looking for small patterns first before considering global ones.

With DL, there is no need for manual feature selection, such as preselection of a set of possible shapes to evaluate in the data, as this step is performed automatically. A DL model coupled with a training module is often called a DL method (Fig. 1B).

### How is DL applied to bioimages?

**Fig. 2.**
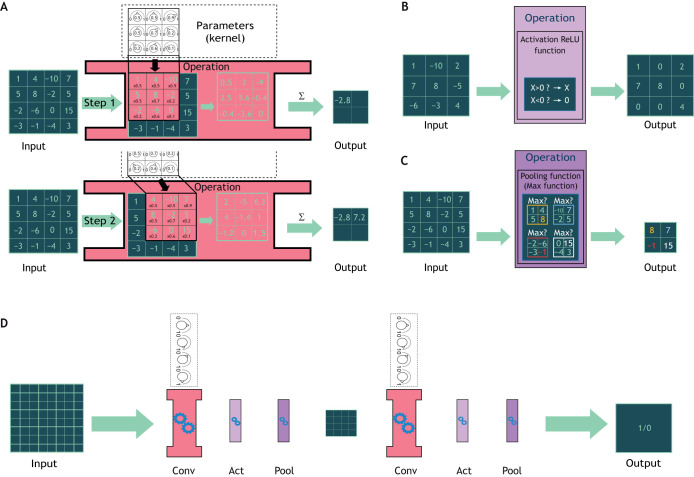
**Examples of DL layers and a CNN workflow for image analysis.** (A) A convolution layer (in red) defined by a 3×3 kernel is applied to a 4×4 input image by a ‘sliding window’ process starting from the top left corner and ending in the bottom right corner. For each new location of the kernel, the weight values are multiplied with the pixel values at this position before being summed (Σ). The result of the sum (−2.8 in step one) is stored in an output image, and the kernel is moved forward to the next step. (B) An activation layer (light purple) decides which regions of the image are important to keep. In this example, a Rectified Linear Unit (ReLU) activation keeps only positive values. (C) A pooling layer (purple) reduces the input dimension, for example as shown here by splitting the image into 2×2 sections and only gathering the maximum value of each of these. (D) A CNN is a succession of sets of convolution (red, Conv; as shown in A), activation (light purple, Act; as shown in B) and pooling layers (purple, Pool; as shown in C). In the example shown here, an 8×8 image, such as an image of a nucleus, goes through a first set of layers and is transformed into a 4×4 feature map, before passing through a second set with the final output a single digit, either 1 or 0, where a value of 1 could mean ‘mutant nucleus’ and a value of 0 could mean ‘wild-type nucleus’.

Most of the DL methods for bioimage analysis are based on a specific type of trainable layer called the convolution layer (Fig. 2A), which gives its name to CNNs. The idea behind the convolution operation is to compute the weighted sum of a small set of pixels that belong to a small sub-window (usually 3×3 pixels) within the input image. The set of weights is called a kernel. This process is reiterated throughout the entire image and aggregated into a new multi-dimensional image. In order to obtain a low-dimensional output, as a single digit, and to force the model to synthesize information, pooling layers have been designed to shrink the dimension of their input by extracting only a chosen set of pixels (Fig. 2C). A succession of several convolution, activation (Fig. 2B) and pooling layers forms the DL model (Fig. 2D). The image is inputted on one side, then sequentially reduced by the pooling layer to obtain a single digit. Such a model could be trained for image classification, which is considered to be the simplest problem in image processing (Fig. 1C). To tackle different problems, such as object localization or segmentation, the classification model is adapted by the addition of more layers and by pre-processing and post-processing (see Glossary) the data before and after the model transformations. For instance, upsampling layers double the size of an input image. Such layers could be sequentially added to the end of our previous nucleus classification model to transform its low-dimensional output into an image the same size as the nucleus image and where each pixel value is either 1 or 0, with 1 (white) for a background pixel and 0 (black) for a pixel of the nucleus (Fig. 1B). This type of output is called a segmentation mask (see Glossary).

The initial model is considered to be a backbone to which specification modules are added to provide specialization (for example detection, segmentation or denoising). Common backbone models include VGG (Simonyan and Zisserman, 2014 preprint), ResNet (He et al., 2016), and EfficientNet (Tan and Le, 2019).

### How are DL methods developed and shared in biology?

**Fig. 3.**
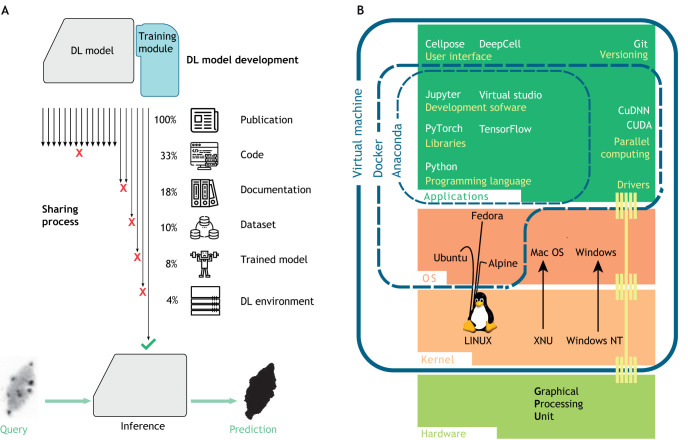
**Sharing and use of DL models.** (A) Necessary elements for DL method sharing. A DL method (top) must provide all the required elements to be reusable (bottom). Despite the profusion of published methods, only 4% have all required components for nucleus segmentation (i.e. four of the 101 references cited in Table S1). (B) Development environment. The development and use of DL methods require powerful hardware resources and dedicated applications (green boxes). Because of incompatibilities between tools and library versions, a proper environment is needed to efficiently share a new method. A good practice for method sharing consists of gathering the configurations and the versions in a dedicated environment (blue rectangles). Such an environment can be built with a package manager, such as Anaconda or Docker, if conflicts occur with the operating system (OS; orange) occur. A virtual machine completely isolates the required configuration but might reduce the available computational resources.

Sharing a DL method is more difficult than sharing non-trainable methods or shallow ML methods (Fig. 3A). To be shared, a DL model should be delivered together with: (1) a detailed explanation in the associated publication; (2) commented code comprising the complete model and its pre- and post-processing steps; (3) documentation containing at least the installation steps and the prediction procedure; (4) a DL environment, including the software and hardware requirements, with the former ideally packaged using Anaconda or Docker (Fig. 3B; Box 1); and, finally, (5) a trained model in the form of a file containing all of the model parameters after training.

**Box 1. Programming tools used for DL**

**Code**. Most DL methods are coded using the easy-to-use programming language Python, but its lack of performance sometimes forces the use of lower-level languages such as C++. DL frameworks, such as TensorFlow, PyTorch, JAX (https://github.com/google/jax), Flax (https://github.com/google/flax) or fastai (https://www.fast.ai/), contain pre-made functions, which facilitate DL coding. For image analysis studies, higher-level frameworks are available, such as TensorFlow model (https://github.com/tensorflow/models), TensorFlow Hub (https://tfhub.dev/), torchvision (https://pytorch.org/vision/stable/index.html), mmDetection (https://github.com/open-mmlab/mmdetection), segmentation_models (https://github.com/qubvel/segmentation_models) or detectron2 (https://github.com/facebookresearch/detectron2). For biomedical images, frameworks such as MONAI (https://monai.io/), nnDetection (https://github.com/MIC-DKFZ/nnDetection), YAPiC (https://yapic.github.io/yapic/) or Gunpowder (https://github.com/funkey/gunpowder), or business solutions, such as Nvidia Clara Imaging (https://developer.nvidia.com/clara-medical-imaging), Microsoft Project InnerEye (https://www.microsoft.com/en-us/research/project/medical-image-analysis/) or Aivia (https://www.aivia-software.com/), can help the analysis. For the specific problem of nuclear image analysis, the best development tools are DeepCell and Cellpose.

**Sharing**. All new developments of DL tools for image analysis are based on existing frameworks, each having an appropriate version and dependencies. A good practice is to package them all into a development environment manager, such as Docker or Anaconda (Fig. 3B). These managers facilitate code sharing and reproducibility. A packaged code is then easy to benchmark, if integrated in a platform such as BIAFLOWS (https://biaflows.neubias.org/), or to share on easy-to-use platforms for biologists, such as Bioimage.io.

**Computational resources**. As DL requires a lot of computational resources to work, both for training and inference, many large companies offer cloud computing services to run code online on their computers. Online DL development can also remove the need for computer configuration by integrating most of the necessary tools. Google Colab (https://research.google.com/colaboratory/), for instance, offers free access to powerful GPUs and allows any user to run Python code online with minimal setup. ZeroCostDL4Mic (https://github.com/HenriquesLab/ZeroCostDL4Mic) is a set of implementations of DL methods for microscopy running in Google Colab. Other competitors are Amazon Web Services (https://aws.amazon.com/machine-learning), Microsoft Azure (https://azure.microsoft.com/en-us/services/machine-learning/) and Paperspace.com (https://www.paperspace.com/).

With these elements available, a DL model can be used to infer predictions. However, to reproduce the DL method (i.e. to train the model or fine-tune it) additional elements are required, such as training and testing datasets***,*** the code of the training module (including the loss, metrics and optimizer definition with all the hyperparameter values), the training routine (i.e. the training pre-processing and post-processing), and additional documentation of the training procedure and setups. Furthermore, to make a tool accessible for those not familiar with programming, the sharing of the inference procedure has to be complemented with an interface, either a command line interface (CLI) or a graphical user interface (GUI). Aside from the datasets (see below), sharing this information can be done on code-sharing platforms, such as GitHub or GitLab**,** or environment-sharing platforms such as DockerHub.

## DL needs large datasets

This section aims to help users in creating a dataset or using an existing one for training or pre-training. We first describe and categorize existing datasets for nuclear image analysis, before listing tools for data labelling. Finally, we focus on data storage and sharing, and discuss the handling of large bioimages.

### Datasets for nuclear image analysis

#### What is a dataset for image analysis?

A dataset is generally a set of pairs of images and their associated annotations. The latter are either scalar annotations (e.g. 0 or 1) for image classification, a set of bounding box coordinates for object detection, or masks for image segmentation. Developers should pay attention to the validity of each pair, because a single mistake may slow down the training process. For example, a mask with the same size, colour depth and format as the original image might be required for each image annotation. Annotations are usually manual and highly time-consuming to make, which should not be underestimated when planning a DL project.

#### Importance of data

Data are the exclusive source of knowledge for the model. Thus, a good dataset must contain all the required information for the model to understand the objects of interest. For instance, does an abrupt change in colour intensity in a piece of an image represent an edge? Are such pixels signal or noise? Do certain objects have regular shapes? Pre-training on a large set of general images may give the model the basis on which to function. Another solution is to manually provide the model with additional information, such as object shape. The StarDist nucleus segmentation method (Weigert et al., 2020), for instance, instructs the model to look for 3D star-shaped polyhedrons, because a nucleus can be assimilated to an invaginated ellipsoid, which can be modelled as a star-shaped polyhedron.

A list of available datasets for 2D and 3D nuclear image analysis is presented in Table 1. These datasets have already been manually segmented and can be used for pre-training. As manual annotation of bioimages is time-consuming, recent approaches have also involved artificially generating pairs of images and annotations by either using a non-DL method such as CytoPacq (https://cbia.fi.muni.cz/simulator/index.php; Wiesner et al., 2019), which has been used for the generation of 3D nucleus images with their segmentation masks, or a DL method, such as that reported by Fu et al. (2018) (Box 2).

**Box 2. Methods for insufficiently labelled datasets**

Four types of solution have been designed to overcome the lack of manually annotated datasets.

**Synthetic data generation.** This approach can be used to complete the training set either by data augmentation or by generative methods. Data augmentation involves applying small transformations to the input image and eventually to the annotation, such as rotations or noise (https://github.com/MIC-DKFZ/batchgenerators), thus providing a new point of view. Generative methods artificially create novel images. Generative Adversarial Networks (GANs; Goodfellow et al., 2014) are DL methods that have been successfully applied to image generation of 3D nuclei (Dunn et al., 2019; Fu et al., 2018), owing to a derivative of the CycleGAN model (Zhu et al., 2017) simultaneously training a nucleus segmentation model.

**Weak supervision.** This approach involves the training of models using noisy or partial annotations (https://github.com/jeromerony/survey_wsl_histology). For instance, annotations with only the nucleus centroid labelled (https://github.com/huiqu18/WeaklySegPartialPoints; Qu et al., 2020) or only the nucleus bounding box added (Zhao et al., 2018) can be used to train the model to perform a full nucleus segmentation.

**Active learning**. Also known as ‘human-in-the-loop’, this method incorporates a human annotator during the training process. While training starts with just a few annotations, the model repeatedly asks a human annotator to label the images that would increase its performance the most. Active learning has already been applied to medical image analysis (Budd et al., 2021), 2D nucleus classification (Shao et al., 2018) and 2D nucleus segmentation (https://github.com/vanvalenlab/deepcell-label; Greenwald et al., 2021).

**Transfer learning.** This approach consists of first giving a general knowledge to the DL model, by, for instance, training it on a large annotated generic dataset, such as ImageNet (Deng et al., 2009) before specializing it by replacement or addition of trainable layers (Kolesnikov et al., 2020). Self-supervised learning is another related solution using large unlabelled datasets to perform pre-training on a so-called pretext task, such as solving the jigsaw puzzle of a split image, before specialization on a so-called downstream task, such as nucleus segmentation in 2D (Buchholz et al., 2020; Sahasrabudhe et al., 2020).

**Table 1.**
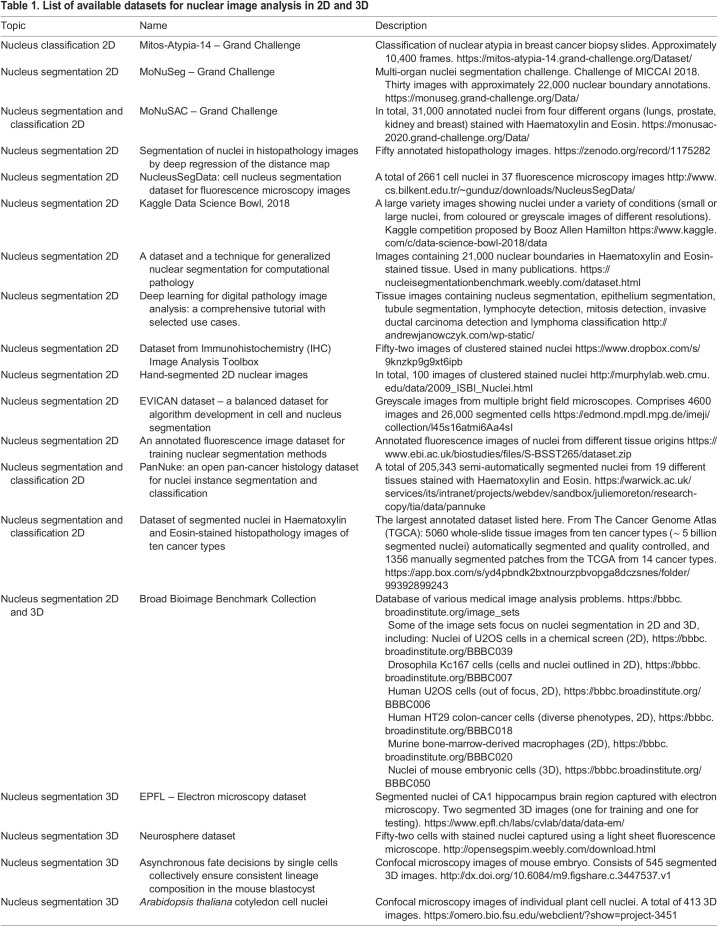
List of available datasets for nuclear image analysis in 2D and 3D

### Data labelling and visualization tools

#### Data annotation and/or labelling

Creating a training dataset requires a good labelling tool. Choosing one depends on the 2D or 3D dimensions of the images and is guided by the imaging application. Here, we present free-to-use tools.

For classification, two pieces of information are extracted from each image and stored in a file: the image name or identifier and its class. 3D bioimages might be classified slice by slice. The ImageJ plugin Qualitative Annotations (Thomas et al., 2021) is an annotation tool for classification, storing the manual annotations in a text file. This plugin relies on the ImageJ selection tools and can also be used for object detection by drawing bounding boxes around objects. 3D Slicer (https://www.slicer.org/), which is designed for annotation of 3D biomedical images, or more general-purpose software, such as VGG Image Annotator (VIA; https://www.robots.ox.ac.uk/~vgg/software/via/; Dutta and Zisserman, 2019) or LabelImg (https://github.com/tzutalin/labelImg) for 2D images and 3D-bat (https://github.com/walzimmer/3d-bat; Zimmer et al., 2019) for 3D images, can also be used. A plethora of solutions exist for the central bioimaging problem of segmentation. 3D Slicer or ITK-SNAP can be used for both 2D and 3D images. The best annotation tools for segmentation we have tested are Ilastik (Berg et al., 2019) and Weka (Arganda-Carreras et al., 2017); both integrate ML algorithms (Random Forest) to quickly label a large number of large 3D images. The webKnossos (https://webknossos.org/; Boergens et al., 2017) platform provides many tools for manual online segmentation in both 2D and 3D.

#### Volume rendering

Visualizing the results of segmentation might be difficult using a slice-only viewer, and 3D Slicer, ITK-SNAP and ImageJ all have integrated tools for volume rendering. Tools such as the Medical Imaging Interaction Toolkit (MITK; Wolf et al., 2004) and Icy (De Chaumont et al., 2012) also incorporate well-adapted visualization features for 3D medical images and bioimages. Napari (Sofroniew et al., 2022) is also a good alternative, because it provides an easy-to-use programmable graphical interface for Python, a language that is widely used in DL programming. Finally, ParaView (Ahrens et al., 2005), which is designed for scientific visualization and volume rendering, allows the user to highlight object structures by applying a range of colours to 3D images using simple thresholds.

### Data sharing

To develop novel DL methods, datasets with their metadata (for example, image modality and resolution) should be made available. The images should be identifiable by their filenames and be in a unified image format. Image quality criteria, such as a good signal-to-noise ratio, a proper image annotation and a limited heterogeneity between objects, should be considered, as outliers can introduce unwanted bias during training.

Such formatted datasets can then be shared through diverse methods, depending on time investment and dataset size. Most of the datasets listed in Table 1 are shared on authors' websites, but ideally a dataset should be stored in a public repository and the associated code should be made available at an open-access repository such as Zenodo or Github/Gitlab. Another solution is to use a cloud-storage platform, which for security reasons, we do not recommend. For bioimages, we recommend the Broad Bioimage Benchmark Collection (BBBC) platform (https://bbbc.broadinstitute.org/; Ljosa et al., 2012); the Image Data Resource (IDR; https://idr.openmicroscopy.org/; Williams et al., 2017) developed by OMERO creators (https://www.openmicroscopy.org/omero/; Allan et al., 2012), which can also be installed on any institution server; and webKnossos.

## DL methods for nuclear image analysis

DL methods trained on manually and fully annotated datasets are called supervised methods (see Glossary) and constitute the current standard approach. However, manual annotation of datasets takes time, and methods dealing with insufficiently labelled datasets are becoming one of the major trends in the field of computer vision. In this section, we present various supervised methods and provide more details about the most accessible among them.

The discussion below is based on 151 publications on DL methods for nuclear image analysis that have been published since 2014 (108 of which were published between 2019 and 2021) either in relevant journals or posted on open-access preprint severs, such as bioRxiv and arXiv (Table S1). For additional information about non-nucleus-specific methods, we advise the reader to visit the Papers With Code website (https://paperswithcode.com/sota), where all the state-of-the-art methods for computer vision problems are listed alongside their code.

### Image classification

Image classification consists of sorting images into categories, called classes (Fig. 1C). Nuclear image analysis studies usually focus on 2D histopathology images of human tissues that have been stained with Haematoxylin and Eosin. Unfortunately, apart from one study (Qu et al., 2019a), the 24 other methods we found do not provide code or datasets, drastically compromising reproducibility (Table S1). To our knowledge, no work has been published on image classification of 3D nuclear images.

For classification of 2D nuclei, basic programming skills are needed to use a standard DL framework (Box 1). Tools that do not require programming have also been developed to help users automatically train and fit models to a private training dataset, but unfortunately these tools are not open-source and free, and often only support 2D images. The most well-known are Google AutoML Vision (https://cloud.google.com/vision), Roboflow (https://app.roboflow.com/; Alexandrova et al., 2015), H2O (https://www.h2o.ai/products/h2o-driverless-ai/) and KNIME (https://www.knime.com/community/image-processing), with the first two offering free trials for image classification problems.

### Object detection

Object detection methods can be classified into distance-map-based and bounding-box-based methods (Fig. 1C). We found 31 publications for 2D and 3D nuclei detection with a dedicated DL model (Table S1). Among these, only five provide a valid code with a trained model [StarDist (Schmidt et al., 2018), SP-CNN (Tofighi et al., 2018), KiNet (Xing et al., 2019), NucleiDetection (Valkonen et al., 2020) and QCANet (Tokuoka et al., 2020)], and only one works with 3D images (QCANet). A distance map for nuclei detection is a greyscale image, of the same size as the input image, where white pixels represent the nuclei and black pixels represent the background. Pixels closer to the centroid of the nucleus have a higher intensity and can thus be used to count nuclei. This technique is the main idea underlying all five methods cited above. In this case, the model is generally a 2D or a 3D U-Net model.

Bounding-box methods, such as Faster R-CNN (Ren et al., 2017) or YOLO (Redmon et al., 2016), frame each object of interest either in a rectangle (2D) or in a rectangular cuboid (3D). Although a third of the 31 publications about nucleus detection employ such methods, none of them present the associated code, and new DL models must thus be trained. A code-free solution is to first find a manually annotated dataset of nuclei before using an easy-to-use graphical interface generated for DL model training, such as ZeroCostDL4Mic (https://github.com/HenriquesLab/ZeroCostDL4Mic; von Chamier et al., 2021). A large set of DL models for object detection (for 2D images only), as well as for image segmentation, denoising and virtual staining, have been encapsulated and set up online on Google Colab servers. Once uploaded on a Google Drive, a nucleus dataset can be used to train an object-detection model such as YOLO online. Once trained, the model will be able to detect nuclei in any set of images. For a programmer, another solution is to employ the implementation of the state-of-the-art methods for general object detection in 2D developed by OpenMMLab (https://github.com/open-mmlab/mmdetection).

### Image segmentation

Segmenting an image involves extracting objects of interest (foreground) from the rest of the image (background) by labelling each pixel with an integer, resulting in what is called a mask. For example, a pixel can be assigned the value 0 if it belongs to the background and 1 if it belongs to a nucleus; this is called semantic segmentation (Fig. 1C). The discrimination of nuclei from each other is referred to as instance segmentation. In this case, each pixel of the first nucleus is labelled with 1, each pixel of the second nucleus is labelled with 2 (and so on), and background pixels are labelled with 0. This task is more difficult than semantic segmentation and is usually implied when referring to nuclei segmentation. Instance segmentation makes it possible to study the morphology of each individual nucleus.

We found 101 recent publications reporting DL methods designed for nuclei segmentation (Table S1). Among those, only 35 provide an open-source implementation, and of these, ten can handle 3D nuclei: CDeep3M (Haberl et al., 2018), DeepSynth (Dunn et al., 2019), 3D U-Net (Cicek et al., 2016), QCANet (Tokuoka et al., 2020), Retina U-Net (Jaeger et al., 2020), NuSeT (Yang et al., 2020), StarDist (Weigert et al., 2020), nnU-Net (Isensee et al., 2021), DeepCell (Van Valen et al., 2016), and Cellpose (Stringer et al., 2021). However, only five provide a trained model: DeepSynth, QCANet, NuSeT, DeepCell and Cellpose.

According to the literature, six main difficulties arise in the segmentation of nuclei: (1) nuclei-related difficulties, including variability in cell size, shape and texture among different organs, tissues and cell types; (2) noise-related difficulties, including poor signal-to-noise ratio, background complexity, uneven colour distribution, heterogenous sample preparation and variations in capture conditions; (3) image modality-related difficulties, including variability of images from different devices (2D versus 3D, confocal and electron microscopes, etc.); (4) manual-annotation-related difficulties, including subjectivity of the manual annotator (inter-observer variability), labelling cost and the small size of handmade datasets in biology; (5) method-related difficulties, including inaccuracies and lack of robustness of classical image processing, configuration, design and training of DL methods, interpretation of DL results and prohibitory computational costs for 3D application; and (6) use-related difficulties, including DL tool setup and use both for computer scientists and biologists. A properly designed DL workflow can tackle difficulties associated with nuclei and noise, as long as a good manually labelled dataset has been built.

#### Semantic segmentation

Most of the current methods for semantic segmentation are based on a very popular DL model called the U-Net encoder–decoder model (Ronneberger et al., 2015), which is also known as a Feature Pyramid Network (FPN). The encoder (the descending U-branch) is a standard CNN, such as ResNet (He et al., 2016), DenseNet (Huang et al., 2017) or EfficientNet (Tan and Le, 2019), or a Transformer model (Hatamizadeh et al., 2022); it progressively transforms the input image into a series of five feature maps, each having a progressively lower resolution – hence ‘feature pyramid’. In its original version (Ronneberger et al., 2015), the encoder is four series of convolution, activation and pooling. The low-resolution encoder output is then composed by the decoder (the ascending U-branch) into the final mask, with the same resolution as the input, by progressively upsampling the feature maps four times and, at each level, applying a series of convolution and activation layers. To retrieve more information, each level of the decoder feature pyramid is merged with its corresponding level in the encoder pyramid before the application of the convolutions. A version of U-Net also exists for 3D images (Cicek et al., 2016), and this has been integrated into the ZeroCostDL4Mic project. Alternatives to U-Net-like models are full-resolution models, which retain the original image resolution at every stage of the model (Qu et al., 2019b) but are more computationally demanding. According to the Papers with Code website (https://paperswithcode.com/sota), one of the current best performing 2D semantic segmentation models is HRNet (https://github.com/HRNet/HRNet-Semantic-Segmentation; Sun et al., 2019); however, this model must be adapted for 3D images, which is not a simple task.

#### Instance segmentation

Four conceptually different approaches for instance segmentation can be distinguished. The first approach uses a semantic segmentation model that classifies pixels into ‘nucleus centre’, ‘nucleus border’ or ‘background’ and couples it with a non-DL method to separate the nuclei clusters. The additional ‘nucleus border’ prediction forces the model to leave sufficient space between each nucleus centre so that they can be separated by a non-DL algorithm. A powerful but computationally demanding example in 2D uses 32 U-Net models (Caicedo et al., 2019) (Table S1). Conversely, the method used by DeepCell, called Mesmer, relies on PanopticNet, a DL model designed to predict both nuclei centres and borders (Greenwald et al., 2021) (Table S1). Mesmer predicts the centre and border of each cell, as well as of each nucleus, and tracks cells in 2D images, in 2D time-lapse data, as well as in 3D images.

The second approach is based on predicting a distance map, similar to the one used for object detection, which is then used to discriminate individual objects from each other by detecting each centroid and, in parallel, to predict each segmentation mask. It is used by StarDist (Weigert et al., 2020) (Table S1) for 2D and 3D images. Cellpose (Stringer et al., 2021) (Table S1) has also adapted this technique for 3D images. Here, segmentations are computed slice-by-slice along each of the three axes and then combined to obtain a 3D mask; this technique is called 2.5D segmentation (Angermann and Haltmeier, 2019).

In the third approach to instance segmentation, a subset of the original image containing the object of interest (a nucleus) is defined using the predictions of a bounding-box detection method. Then, a semantic approach is used to segment the isolated nuclei. This is the main concept underlying the Mask R-CNN method (He et al., 2020), which integrates these steps in a single model by specializing an image-classification model with several trainable modules to extract features of different sizes and to obtain object classes, bounding boxes and their individual masks. Mask R-CNN is designed for 2D instance segmentation. An adaptation has been created for 3D images (Jaeger et al., 2020; https://github.com/MIC-DKFZ/medicaldetectiontoolkit) and applied to the segmentation of human tissue images. An adaptation attempt has also been made for the 3D nucleus (Tatout et al., 2022).

The final method is a parallelization of the previous approach; detection of nuclei and semantic segmentation are computed separately with a DL model for the entire image before being combined using a non-DL method, resulting in separate nuclei clusters. The two main methods using this technique for 3D images are QCANet (Tokuoka et al., 2020) and NuSeT (Yang et al., 2020) (Table S1). QCANet uses two different models for bounding-box detection and segmentation, whereas NuSeT uses a single U-Net model to compute both. Additionally, NuSeT provides a user-friendly interface.

To conclude, if planning to segment 2D nuclei, we advise first trying the online version of DeepCell (https://www.deepcell.org/), Cellpose (https://www.cellpose.org/) or NucleAIzer (https://www.nucleaizer.org/), which has a well-designed graphical interface and does not require any setup. However, in case of 3D nucleus segmentation, if previous results are inaccurate, the model must be applied to a large dataset or the data cannot be uploaded to an online server, we suggest using and configuring the offline version of DeepCell (Van Valen et al., 2016), Cellpose (Stringer et al., 2021), QCANet (Tokuoka et al., 2020) or NuSeT (Yang et al., 2020). The setup of these methods remains tedious, and good results are only guaranteed on images that are sufficiently similar to those used to train them. For other images, the model will have to be fine-tuned or retrained from scratch on a new manually labelled dataset. We advise non-programmers to use ZeroCostDL4Mic, which also contains DL models for segmentation, or alternatively either the U-Net (Falk et al., 2019) or DeepImageJ (Gómez-de-Mariscal et al., 2021) ImageJ/Fiji plugins, which both have a graphical interface. The U-Net plugin integrates a pre-trained model for 2D and 3D segmentation, which can be refined using a few images if it is installed with appropriate software on a powerful computer. DeepImageJ works with the Bioimage.io website (https://bioimage.io), where many DL models for image segmentation, as well as for other image analysis problems, can be uploaded to the plugin. These models are currently non-retrainable, but some of the segmentation models created with ZeroCostDL4Mic are compatible with the plugin. For users more familiar with programming, nnU-Net (Isensee et al., 2021; https://github.com/MIC-DKFZ/nnUNet) is recommended for 3D instance segmentation because it automatically handles the full model configuration and training, and for 2D images, implementation of most state-of-the-art methods for segmentation can be found on GitHub (https://github.com/qubvel/segmentation_models.pytorch).

### Image denoising

A better visualization of nuclei, from the point of view of both the human eye and DL, could facilitate image analysis. Denoising has also been explored with the help of DL methods. We have found five methods that provide the underlying code: VoidSeg (Prakash et al., 2020), Noise2Void (Krull et al., 2019), DenoiSeg (Buchholz et al., 2020), DecoNoising (Goncharova et al., 2020) and 3D-RCAN (Chen et al., 2021) (Table S1). They all use a U-Net model by training it with artificially noised images to reproduce the original image. The first three solutions are based on the CSBDeep (Weigert et al., 2018) toolbox (Table S1) and have proven their efficiency with 2D nucleus images. In particular, the DenoiSeg method couples denoising and segmentation in the same model and has demonstrated a substantial improvement in 2D nuclear segmentation compared with other methods, such as U-Net alone or StarDist. In addition, 3D-RCAN is also suitable for denoising 3D images. Programmers might be interested in looking at these methods, whereas non-programmers could employ one of the easy-to-use denoising models integrated into ZeroCostDL4Mic if they have access to a suitable dataset, which only needs to be composed of raw images and does not require manual labelling.

The imaging problems discussed above are currently the main focus of DL methods for nucleus image analysis, but readers might be interested to explore future challenges proposed by the 4D Nucleome initiative (https://www.4dnucleome.eu/).

## Conclusions

DL methods are powerful techniques for image analysis that can reach levels of accuracy not achieved previously and automate the formerly manual feature-selection step. Instead of being required for feature selection, human intervention is now needed for dataset creation and design of DL methods. This shift in focus also needs to be applied to the code-sharing process. If an article reporting a method and its associated code are the DNA of DL, then the datasets, trained model, documentation and development environment can be considered the epigenetic signals required for its proper functioning. Sharing the former without the latter is to give a solution without the means to apply it. DL studies of the 3D nucleus do not avoid this reality, and unfortunately far too many methods are not reusable.

There are several ways by which the gap between biologists and computer scientists could be bridged: firstly, by biologists sharing their datasets publicly to provide valuable resources to DL engineers; and secondly, by computer scientists sharing their methods, including all appropriate material and, ideally, with an easy-to-use interface. Finally, we believe that the best way to bridge this gap is to create interdisciplinary teams and to support good practice in specialized method sharing.

**Glossary**

**Dataset**: a set of raw images and their corresponding manual annotations (also called labels or ground truths). These two sets are usually split into a training set to train the model, and a testing set to assess the performance of the model. Supervised, weakly or semi-supervised, and unsupervised models refer to DL models trained with annotated, partially annotated or unannotated image datasets, respectively.

**Deep learning**: a subset of ML methods that contains a neural network model (also called architecture) with a sequence of more than one trainable layer.

**Feature map**: an intermediate output containing the features extracted by a convolution layer, followed by an activation layer. It usually takes the form of a 3D image (height, width, depth).

**Hyperparameters**: a set of manually defined parameters. Example of hyperparameters are model hyperparameters (e.g. number of layers, their size, their arrangement) or the training hyperparameters (e.g. learning rate, batch size).

**Layer**: a transformation applied to an input (an image or a feature map in our case). It can be trainable (containing parameters and/or weights updated during training) or not.

**Loss function**: a function comparing the ground truth with the model output. The obtained loss value measures the performance of the model and is then used to update the model parameters during training.

**Mask**: a binary image having pixels equal to either zero (black) or one (white). A mask can be created manually or as the output of a segmentation model.

**Post-processing**: a set of operations applied to the model output before computing a prediction or the loss function. Examples of post-processing are sigmoid transform or more complex operations, such as those used in by StarDist (Weigert et al., 2020).

**Pre-processing**: a set of operations applied to the input before being inputted into the model. An example of a pre-processing operation is data augmentation.

**Training**: computation of the loss value followed by updating of the model parameters with the help of an optimizer algorithm that backpropagates the loss through the model.

## Supplementary Material

Supplementary information
