# Supplementary material for: Deep learning ­– promises for 3D nuclear imaging: a guide for biologists
Source: J Cell Sci. 2022 Apr 14;135(7):jcs258986. doi: 10.1242/jcs.258986 (PMC9016621; doi:10.1242/jcs.258986)
Supplement: Supplementary information [file joces-135-258986-s1.pdf]

Table S1. List of all investigated publications presented in the 'DL methods for nuclear image analysis' section.

For each method, we checked whether the following elements are available online: (1) the code, (2) the documentation, (3) the dataset, (4) the trained model and (5) the development environment.

A documentation is judged valid if it is sufficiently detailed and lets a new user quickly understand how to use the code. The dataset can be a publicly accessible dataset but not necessarily associated with the publication. A link toward a trained model file must be provided. The deep learning development environment can be a Python package (or any kind of package depending on the language), a Conda setup or a Docker image. We also mention in the last column whether the method is adapted for 2D or 3D images. Some methods (in grey) are generic methods, which are not adapted specifically to nucleus images but are flexible methods that should be easily adaptable for bioimaging.

Click here to download Table S1
